# A systems level framework for postharvest physiology and quality preservation

**DOI:** 10.3389/fpls.2026.1763497

**Published:** 2026-02-06

**Authors:** María E. García-Pastor, Natalia Falagán

**Affiliations:** 1 Department of Applied Biology, Institute for Agri-Food and Agro-Environmental Research and Innovation (CIAGRO), University Miguel Hernández, Alicante, Spain; 2 Plant Science Laboratory, Cranfield University, Cranfield, United Kingdom

**Keywords:** food loss reduction, fruit and vegetable quality, metabolomics, molecular mechanisms, postharvest biology, ripening and senescence

## Abstract

The postharvest phase is critical for determining the quality, nutritional value, and market viability of fresh produce, yet global losses remain substantial, often exceeding 40%. This perspective aims to establish an integrated framework for understanding postharvest physiology and guiding sustainable quality preservation strategies. Deterioration is driven by complex molecular and physiological transformations, including ripening, senescence, and oxidative stress. Understanding these mechanisms is paramount for developing effective loss and waste reduction strategies. Metabolomics provides a systems level view of these changes, enabling the large scale profiling of small molecules and the identification of valuable biomarkers for quality loss, chilling injury, and senescence. Shifts in primary metabolites (sugars, organic acids) and the accumulation of ‘off aroma’ volatiles (ethanol, acetaldehyde) are critical indicators of decline. Also, preharvest factors (e.g. regulated deficit irrigation, signalling molecule application) fundamentally influence postharvest metabolic states by enhancing antioxidant capacity and delaying senescence. Molecular regulation, orchestrated by hormonal signalling (ethylene, abscisic acid) and transcription factors, underpins these shifts. Interventions focus on sustained redox homeostasis, often achieved through the exogenous application of ecofriendly signalling molecules like salicylic acid to upregulate enzymatic and non-enzymatic antioxidant systems. Integrating multi-omics technologies (metabolomics, transcriptomics) facilitates the identification of molecular targets for these interventions and supports predictive modelling for optimising storage conditions. Translating these integrated insights into sustainable, biomarker based, farm to fork strategies is essential for enhancing food security and mitigating global greenhouse gas emissions associated with food loss.

## 1 Introduction

Optimal postharvest management remains a critical bottleneck to global food security and sustainability. Despite significant advances in research and development, it is estimated that 30-50% of fresh produces are lost between harvest and consumption, leading to negative impacts on the economy, nutrition and the environment (Iori et al., 2023; Gage et al., 2025). These losses contribute to greenhouse gas emissions and undermine food system resilience. Addressing this challenge requires more than improvements in infrastructure implementation; it demands a mechanistic understanding of the biochemical, physiological and molecular processes driving ripening and senescence after harvest and a robust strategy that integrates pre- and postharvest handling.

Fresh produce is metabolically active after harvest, undergoing ripening, senescence, and stress responses influenced by environmental conditions, handling, and intrinsic physiology. These processes are tightly associated with oxidative stress, cell wall disassembly, energy depletion, and shifting hormonal balances that modulate both quality attributes and susceptibility to fungal pathogens. Recent advances in metabolomics and multi-omics now provide unprecedented insight into these processes, enabling the identification of metabolic signatures that predict quality decline, guide intervention design, and support sustainability oriented innovations.

This perspective argues that the future of sustainable postharvest management lies in integrating mechanistic understanding into supply chain decision making. By viewing postharvest physiology as the continuation of a biochemical trajectory shaped in the orchard, the field can move towards predictive, personalised, and resource efficient strategies that minimise losses and enhance produce quality.

We take a systems level framework, which refers to the explicit integration of biological processes across organisational scales, ranging from molecular and cellular regulation to tissue, organ, and whole commodity behaviour, together with decision points along the postharvest supply chain, from preharvest management to storage, distribution, and retail. Rather than treating postharvest deterioration as a set of isolated physiological events or technological interventions, this approach conceptualises quality loss as the emergent outcome of interacting metabolic networks, regulatory pathways, environmental conditions, and management decisions. By linking multi-omics derived biomarkers to actionable interventions and monitoring strategies, the proposed framework enables feedback between mechanistic understanding and practical implementation, supporting predictive and adaptive postharvest management.

## 2 Mapping biochemical changes through postharvest metabolomics

Metabolomics enables large scale profiling of small molecules and offers a direct understanding into the biochemical state of harvested produce (Yun et al., 2022). By mapping dynamic changes in sugars, organic acids, amino acids, and secondary metabolites, the approach reveals the pathways underpinning flavour, aroma, nutritional quality, stress responses and senescence.

Real time measurements of respiration rate and ethylene production together with techniques like gas chromatography mass spectrometry, liquid chromatography mass spectrometry, and nuclear magnetic resonance have enabled detailed characterisation of ripening progression, oxidative stress, and responses to storage environments, including postharvest disorders like chilling injury. These insights have moved the field beyond descriptive physiology toward mechanistic understanding, facilitating the development of targeted interventions. Historically, postharvest research focused on physiological indicators of deterioration, such as firmness loss, colour change, or respiration rate. Metabolomics now enables actionable biochemical markers that can serve as early warning signals or even predictive tools for postharvest behaviour (Pacheco-Ruiz et al., 2025). However, the field must increasingly shift from cataloguing metabolites to integrating metabolic networks with gene expression, enzyme activity, and environmental data to build predictive, scalable tools.

Although metabolomics provides the most direct readout of postharvest physiological status, its explanatory power is enhanced through integration with complementary omics approaches. Transcriptomics elucidates regulatory networks governing ripening, senescence, and stress responses, linking hormone signalling and transcriptional control to downstream metabolic shifts. Lipidomics offers additional insight into membrane composition and lipid peroxidation processes central to chilling injury, oxidative stress, and loss of cellular integrity. Together, these approaches support a systems level understanding of postharvest deterioration beyond metabolite profiling alone.

### 2.1 Biomarker discovery for quality and deterioration

One of the most transformative contributions of metabolomics has been the identification of biomarkers associated with postharvest resilience or susceptibility. Markers of oxidative stress, such as malondialdehyde accumulation, shifts in phenolic or flavonoid profiles, or depletion of ascorbate pools, are closely linked to senescence progression (Dobón-Suárez et al., 2025a). Pathogen susceptibility is similarly associated with transitions in defence related metabolites driven by hormonal trade offs during ripening. The endogenous accumulation of abscisic acid is a validated biomarker for postharvest senescence in non climacteric table grapes, as its peak precedes and correlates with decreased firmness and increased respiration rate and mould incidence (Navarro-Calderón et al., 2023). Cultivar- and tissue-specific accumulation of abscisic acid and the concomitant ethylene production burst are key metabolic biomarkers strongly correlated with fruit softening and over-ripening during the postharvest supply chain (García-Pastor et al., 2021a).

Primary metabolite patterns, such as changes in sugars (glucose, fructose, sucrose) and organic acids (malate, citrate), have proven essential for predicting flavour evolution (Pott et al., 2020), chilling injury, and firmness. For example, malate levels correlate with firmness retention in tomatoes and other fruit, offering a promising trait for breeding programmes.

The predictive value of metabolite biomarkers is strengthened when supported by transcriptomic or lipidomic evidence. Expression patterns of key biosynthetic enzymes, antioxidant systems, and hormone related genes frequently mirror metabolite accumulation, while lipidomic markers of membrane degradation complement oxidative stress indicators. Integrating these molecular layers enhances biomarker robustness and predictive accuracy.

Compatible solutes like proline, trehalose, raffinose, and galactinol serve as osmoprotectants and reactive oxygen species (ROS) scavengers and have emerged as markers of chilling tolerance in grapes, plums, and citrus. Secondary metabolites such as α-farnesene have shown strong associations with storage disorders, reinforcing the value of metabolite based diagnostics (Pott et al., 2020).

Biomarker discovery is increasingly robust, but the next step lies in predictive modelling and real time decision support. This will require:

▪ large scale validation across cultivars and environments.
▪ integration with phenomics and environmental monitoring. and
▪ development of low cost sensors capable of detecting metabolite signatures from farm to fork.

Such developments could transform postharvest management from reactive to predictive, enabling dynamic adjustment of storage conditions based on physiological state rather than fixed protocols.

### 2.2 Linking preharvest conditions to postharvest metabolism

Postharvest outcomes are not solely determined after harvest; they begin in the field. Nutrient availability, irrigation strategy, canopy microclimate, hormonal balance, and exposure to abiotic stress collectively shape fruit biochemical composition at harvest. This initial state governs respiration rate, ethylene biosynthesis, antioxidant capacity, susceptibility to disorders, and response to storage conditions.

We now know that nutrition and mineral balance are key determinants of storage potential. For example, calcium availability fortifies cell walls and membranes, reducing disorders such as bitter pit. Excessive nitrogen, in contrast, accelerates ripening, increases ethylene production, and reduces storage life. These dynamics underscore the need for nutrient management strategies aligned with intended storage duration.

Regarding water management, previous studies have shown how irrigation regimes modulate stress response metabolism, carbohydrate accumulation, and peel integrity. Mild, well timed deficit irrigation can enhance antioxidant capacity and delay senescence, whereas severe stress increases disorder risk. Environmental stresses, such as heat or solar radiation, similarly imprint stress signatures that persist into storage.

Preharvest signalling molecules can also act as metabolic primers. Applications of salicylic acid, oxalic acid, abscisic acid, or ethylene modulating bioregulators have shown promise in enhancing antioxidant systems, modulating ripening, or improving colour and flavour development. These treatments can ‘prime’ fruit for improved postharvest resilience by altering gene expression and metabolic processes.

While growing evidence demonstrates preharvest influences on postharvest performance, far fewer studies quantify consequences for energy use, storage duration, cooling requirements, or greenhouse gas emissions. Integrating metabolomics with life cycle assessment represents a critical future direction, enabling orchard management decisions to be evaluated with their full environmental implications.

## 3 Sustainable strategies to reduce postharvest losses

### 3.1 Molecular and biochemical interventions

Molecular interventions aim to modulate the biochemical processes underpinning deterioration. They constitute a sustainable paradigm shift aimed at reducing postharvest losses by precisely manipulating the physiological mechanisms driving deterioration, primarily focusing on ripening, senescence, and stress response pathways (Chowdhary and Alkan, 2025). Ethylene suppression (e.g. use of 1-methylcyclopropene) remains commercially dominant, delaying ripening and reducing respiration rate (Watkins, 2006). However, sustainable alternatives are emerging such as i) priming with natural elicitors (e.g. salicylic acid, methyl jasmonate, oxalic acid), that enhances antioxidant systems and induces defence related genes; ii) manipulation of hormonal pathways, including abscisic and jasmonic acids signalling, which can delay senescence or improve stress tolerance; and iii) redox homeostasis modulation that helps maintain cellular integrity under storage stress.

The future lies in integrated biochemical strategies that complement preharvest interventions and are supported by metabolomics driven diagnostics. The efficacy of molecular interventions is increasingly evaluated using integrated multi-omics frameworks. Transcriptomics captures treatment induced reprogramming of stress, defence, and redox related genes, while lipidomics reveals stabilisation of membrane integrity under storage stress. Combined with metabolomics, these approaches enable the optimisation of intervention strategies.

### 3.2 Biomarker based monitoring and smart storage

Static storage conditions, including fixed temperature, humidity, and gaseous environment, do not account for the dynamically changing physiology of fresh produce. As metabolomic biomarkers mature, their incorporation into real time monitoring technologies offers a path toward adaptive storage. Electronic noses, near-infrared spectroscopy, or metabolite biosensors can detect volatiles or key metabolites signalling early deterioration, enabling dynamic adjustments of storage conditions to minimise losses.

The integration of these metabolite markers, often derived from multi-omics analysis, into real time monitoring technologies allows for the crucial move away from traditional, static management practices (Gage et al., 2025). This capacity for prediction facilitates the dynamic adjustment of storage conditions (e.g. optimising temperature or atmospheric composition) based on the current physiological state of the produce, which is essential for optimising postharvest protocols, maintaining quality attributes, and mitigating substantial waste risks. Ultimately, real time physiological monitoring will be central to future sustainable cold-chain systems.

From a systems perspective, the effectiveness of packaging and alternative options such as edible coatings is enhanced when informed by physiological state rather than applied as fixed solutions. Integrating metabolomic biomarkers and sensor based monitoring with atmosphere management enables dynamic adjustment of gaseous composition and storage conditions in response to real time metabolic signals, reducing the risk of anaerobic metabolism, off-flavour development, and physiological disorders. In this way, packaging and atmosphere control are not standalone technologies but responsive components of an integrated, biomarker guided postharvest system.

### 3.3 Integrated pre and postharvest management

Sustainability in postharvest systems cannot be achieved through postharvest innovation alone. Aligning field practices with postharvest management goals creates a continuum of quality and safety preservation. Regulated deficit irrigation, targeted nutrient strategies, and preharvest elicitor treatments must be paired with metabolomics guided postharvest protocols and responsive storage systems. Integrated pre- and postharvest management combines upstream agronomic precision with downstream molecular diagnostics, establishing a cohesive farm to fork strategy crucial for sustainability and mitigating substantial global food loss and waste. Preharvest factors, such as irrigation management and nutrient availability, fundamentally influence the initial quality and subsequent postharvest metabolic trajectory of fresh produce (Gage et al., 2025). Regulated deficit irrigation serves as a successful field practice, enabling water conservation while simultaneously enhancing the concentration of health promoting compounds and inducing the activity of oxidative stress mitigating enzymes, such as catalase and ascorbate peroxidase, in fruits like early peaches (Falagán et al., 2016).

Complementary strategies involve the preharvest application of environmentally friendly elicitors or signalling molecules to induce innate defence mechanisms, thereby molecularly priming the produce for postharvest stress (Dobón-Suárez et al., 2025b). For example, preharvest application of salicylic acid enhances fruit antioxidant capacity, delays senescence, and improves postharvest quality in crops like lemon and green pepper (Serna-Escolano et al., 2021; Dobón-Suárez et al., 2021; 2025a). In green pepper, irrigation proved to be an effective salicylic acid delivery method due to its ease of applicability and cost effectiveness (Dobón-Suárez et al., 2025a). Similarly, preharvest oxalic acid application in table grapes was found to improve colour and quality by stimulating the antioxidant system and upregulating abscisic acid metabolism, including *Vv*NCED1 gene expression (García-Pastor et al., 2021b). Finally, preharvest foliar application of the eco-friendly signalling molecule 24-epibrassinolide successfully molecularly primed ‘Sanguinelli’ blood oranges, resulting in a significant boost in crop yield and enhanced commercial quality attributes, particularly red colour (anthocyanin content) and firmness (Garrido-Auñón et al., 2025).

The optimisation of these integrated strategies relies on multi-omics technologies and advanced analytical diagnostics (e.g. metabolomics) to provide comprehensive, systems level mechanistic insights into the complex interactions between genes, proteins, and metabolites, thereby enabling predictive quality models and dynamic adjustments necessary for enhancing postharvest resilience and efficiency (Habibi et al., 2024; Gage et al., 2025).

## 4 Challenges and future directions

Despite progress, several challenges must be addressed to realise a fully predictive, sustainable postharvest system.

1. Biological variability: Responses differ across cultivars, environments, and storage conditions, complicating universal biomarker identification (Gage et al., 2025).
2. Causality vs. correlation: Functional validation of gene–metabolite–phenotype relationships is required.
3. Standardisation and reproducibility: Consistent protocols for sampling, metabolite extraction, and data processing are needed.
4. Translational adoption: Developing cost effective, scalable tools for industry application remains critical.
5. Capacity building: Enhancing expertise in omics analysis and interpretation, particularly in resource limited contexts, is essential.
6. Cellular resolution: Bulk tissue analyses may mask spatial and cellular heterogeneity in postharvest responses. Emerging single cell and spatial transcriptomics offer new opportunities to resolve cell specific regulatory mechanisms and refine biomarker discovery when integrated with metabolomics.

**Figure 1:**
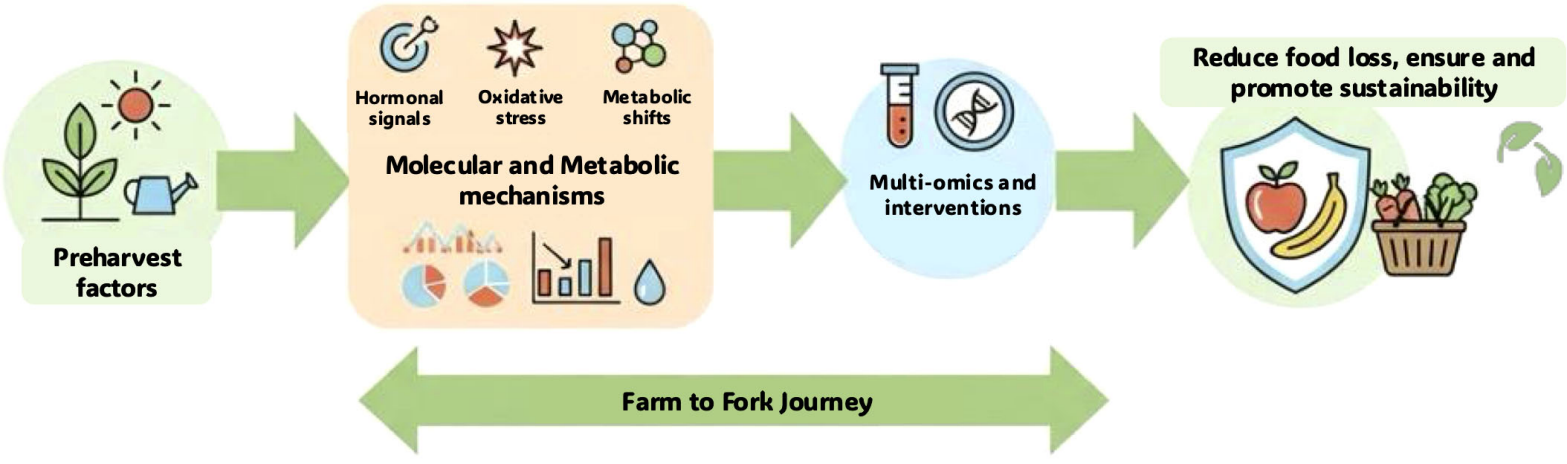
Integrated farm to fork strategy: Leveraging molecular mechanisms and multi-omics for sustainable food loss reduction.

Addressing these challenges will require interdisciplinary collaboration, large scale data integration, and translational research bridging molecular insights with practical solutions. Sustainability metrics will need to be embedded into biological research (**Figure 1**). It is also interesting to highlight the role of standardisation and reproducibility for translating metabolomics and multi-omics insights into predictive postharvest tools. Adoption of established minimum reporting standards for metabolomics (Sumner et al., 2007), together with adherence to FAIR (Findable, Accessible, Interoperable, and Reusable) data principles (Wilkinson et al., 2016), is essential to enable cross-study comparison, data integration, and validation. In practical terms, this requires comprehensive reporting of experimental design, sampling protocols, analytical methods, and associated metadata to ensure that datasets can be reused and meaningfully compared across commodities, environments, and storage systems.

Metabolomic and molecular approaches have transformed our understanding of postharvest physiology in fresh produce. By elucidating the biochemical pathways and regulatory networks underlying ripening, senescence, and stress responses, these tools enable biomarker discovery and identification of molecular targets for intervention. Translating these insights into practical strategies, including predictive monitoring, targeted treatments, and integrated pre- and postharvest management, can prolong shelf life, enhance quality, and reduce food loss. Advancing multi-omics integration and fostering translational research will be integral for developing sustainable, high quality fresh produce systems that meet global food security and nutrition challenges.

The next decade offers a profound opportunity: to transform postharvest management from a reactive discipline into a predictive science powered by metabolomics, multi-omics integration, and smart technology. If successfully realised, this shift will contribute to more resilient food systems capable of meeting global nutrition and sustainability challenges.

## Data Availability

The original contributions presented in the study are included in the article/supplementary material. Further inquiries can be directed to the corresponding author.
